# Medical students’ attitude towards psychiatry: a comparison of past and present

**DOI:** 10.1038/s41598-023-35797-y

**Published:** 2023-05-29

**Authors:** Punjaree Wiriyacosol, Awirut Oon-arom, Chawisa Suradom, Nahathai Wongpakaran, Tinakon Wongpakaran

**Affiliations:** 1 Department of Psychiatry, Faculty of Medicine, Chiang Mai University, Chiang Mai, 50200 Thailand; 2 Psychotherapy Unit and Geriatric Psychiatry Unit, Department of Psychiatry, Faculty of Medicine, Chiang Mai University, 110 Intawaroros Rd., T. Sriphum, A. Muang, Chiang Mai, 50200 Thailand

**Keywords:** Psychology, Medical research

## Abstract

Attitude to psychiatry influences motivation for medical students to successfully achieve in studying psychiatry. With a new generation of students, it would be interesting to investigate how attitudes have changed. This study aimed to compare the attitude of fifth-year medical students toward psychiatry in recent and in the past 24 years. Two samples of fifth-year medical students at Chiang Mai University completed the 30-item attitude to psychiatry (ATP-30); 118 students completed it in 1996, whereas 242 medical students completed it in 2019. Rasch analysis was employed for examining the differences between the total score and individual item scores between the two groups. The total score of ATP in the 2019 group was significantly higher than that in the 1996 group. After misfitting individuals and biased items were removed, only 15 items were valid and useful for a comparison. Of 15 items, 11 were found highly significantly different between two groups (p < 0.001). Negatively worded items, e.g., no strong evidence indicating effectiveness, became easier to score items (increased positive attitude) whereas some positively worded items, e.g., I would like to be a psychiatrist, became more difficult (less positive attitude) comparing between 1996 and 2019. In a comparison between the two methods using the traditional t-test and Rasch analysis, only 5 of 30 items (16.7%) agreed with each other. The overall attitude to psychiatry was significantly higher at the present compared with that in the past. Most items did not differ between the two times. Further studies regarding improving the attitude scale using item response theory such as Rasch should be encouraged.

## Introduction

A survey in 2017 revealed that Thailand has 886 psychiatrists for a 65.3 million population (1.4: 100,000)^1^. This indicates that a much greater number of psychiatrists are needed. The Thai residency training program in psychiatry was established in 1969, meaning that we have new 17 psychiatrists on average each year. Even though the number of doctors applying for positions in the psychiatry residency training program is higher at present than in the past, it remains insufficient compared with the population. In addition, psychiatry requires suitable attributes of the applicant as well as the right attitude for training because psychiatry is a unique subspecialty compared with others in medicine. In most medical schools in Thailand, medical students have a psychiatry clerkship for four weeks in the fifth year, which is the appropriate time to learn about this subspecialty. Unlike other branches of medicine, psychiatry focuses on psychosocial etiology and treatment. It takes medical students quite some time to adjust their cognitive approach from a medical model to a bio-psycho-social model. Uncertainty, fear, lack of confidence, and doubts related to psychiatry are usually abolished or mitigated after exposure to the clerkship.

A review from 22 different countries collecting 12,144 students’ opinions supported that the majority found overall student attitudes were quite positive^2^. Related studies have shown that overall medical students reported an “above neutral attitude” on average (score > 90)^3,4^. The impact of the length of clerkship exposure on the attitudes toward psychiatry is controversial. Some studies showed that “more than one month” of exposure to psychiatry showed a better attitude compared with the “one month or less” option^5^, whereas some revealed that no significant change in the Attitude to Psychiatry (ATP) total score was noted between before and after a short term (4 weeks or less) clerkship^6^. Dating back to 2003, a study conducted in southern Thailand showed that the average score implied a more favorable attitude (mean = 110, SD = 10.28). However, there were no significant differences in ATP scores comparing before and after the 4 weeks of psychiatric clerkship^7^. Several factors related to the clerkship experience could have contributed to the results, such as the duration of exposure, attending staff, and learning opportunities available during the specific period.

Still, attitude towards psychiatry is important for studying psychiatry and psychiatry training even though the decision to choose psychiatry training may depend on many factors such as teaching methods, quality and length of clinical exposure, electives, and enrichment activities, an opportunity to make money, preference in psychiatry as well as personal traits and personal experience related to mental illness, e.g., students experiencing a family history of mental illness tended to score significantly higher with positive attitudes toward psychiatry^8^.

In the past decade, the growth of neuroscience was spurred by the discovery of new neuro-imaging techniques such as functional magnetic resonance imaging (fMRI) which allowed a better understanding of brain activities and pathologies. From 1990 to 2011, the probability that a brain disease was endorsed as a possible cause of schizophrenia increased significantly, whereas the probability of negative life events being a cause decreased slightly^9^. Such findings underscore that schizophrenia is more likely to be related to the brain rather than mental disorders, entailing a favorable attitude towards psychiatry as one part of science.

Students should have more opportunities to accept psychiatry as a science due to its more advanced neuroscience. The Diagnostic and Statistical Manual of Mental Disorders (DSM) has fundamentally shaped nearly all aspects of psychiatric perspectives and practice. In 1994, the fourth edition (DSM-IV) was published stating that physicians should not imply any fundamental distinction between mental disorders and general medical conditions and that all DSM mental disorders were at least partly organic. Since the DSM-IV, strict standards of scientific evidence from the latest research were required for introducing new diagnoses. The latest fifth edition (DSM-5) changed methods of assessment to a clearer approach and improved clinicians’ ability to identify diagnoses^10^. The DSM also destigmatized mental conditions by modifying terminologies such as mental retardation to intellectual disability, substance addiction to substance dependence to substance use disorder, social phobia to social anxiety disorder, and gender identity disorder to gender dysphoria. These changes may make psychiatry less stigmatized and more acceptable. In addition, high-speed internet, and ubiquitously accessible search engines especially via smartphones have greatly impacted acquiring knowledge regarding mental health and psychiatry. This provides students with a vast opportunity to learn about psychiatry at their will.

The psychiatric curriculum in the Faculty of Medicine, Chiang Mai University has changed over 20 years. Before 2005, a four-week psychiatry clerkship began in the fifth year after completing major fields of internal medicine, surgery, OB-GYN, paediatrics, and orthopaedics). From the authors’ observation, students’ attitude to psychiatry was notably higher in post-clerkship. The year of 2005 to 2010, the clerkship was split into two weeks rotations in the fourth and fifth years. That did not help improve attitudes. From 2010 to the present, a total of 4 weeks’ clerkship in the fifth year was brought back and the attitude towards psychiatry increased after the clerkship as before.

The state-of-art technology to access knowledge at present supports the fact that recent medical students constitute a new generation. Exploring their attitudes towards psychiatry after their psychiatry clerkship would be interesting compared with those in the past two decades. Therefore, the authors examined and compared the recent post-clerkship attitude towards psychiatry of medical students and the past using the same measurement hoping to see how they changed over two decades. With the high accessibility to knowledge at present, we hypothesized that recent medical students would have a higher level of attitude toward psychiatry than those in the past. The items regarding understanding and knowledge should be higher, and the students’ desire to be psychiatrists should also be higher than that in the past based on such increased knowledge and understanding.

## Materials and methods

This research employed a retrospective, cross-sectional design using data from 1996 and 2019 for analysis. The study was conducted according to the guidelines of the Declaration of Helsinki and approved by the Institutional Review Board (or Ethics Committee) of the Faculty of Medicine, Chiang Mai University.

### Participants

Of 128 students from the academic year of 1996, 118 (92.2%) participated in the survey. Of 282 students in the year of 2019, 242 (85.8%) participated in the survey. All 360 completed the attitude to psychiatry scale (ATP-30) at the end of clinical clerking in the fifth year of medical education.

### Instrument

#### Attitude to psychiatry (ATP-30)

The ATP-30, developed by Burra et al.^11^, is a 30-item tool in the format of a five-point Likert scale, ranging from strongly disagree (1) to strongly agree (5). The ATP-30 has eight categories addressing psychiatrists, psychiatric knowledge, psychiatric career choice, psychiatric patients, psychiatric disorders and treatment, psychiatric institutions, and psychiatric teaching. The ATP-30 contains both negative and positive statements, for example, “It is quite easy for me to accept the efficacy of psychotherapy” and “There is very little that psychiatrists can do for their patients.” A total score is calculated by summing up all 30 items after all negative items have been reversed (minimum score 30, maximum score 150). A high number indicates a more positive attitude.

### Statistical analysis

Demographic data were described using mean, standard deviation (SD), and frequency. T-test was used for total score comparison. Mann–Whitney U test was used to identify the significant difference between the item score of both times. A p-value < 0.05 was considered statistically significant.

To compare two groups, the biased items or differential item functioning (DIF) should be excluded. Rasch analysis provides a powerful method for detecting and understanding DIF in test items and can help ensure that test scores are valid and fair for all test-takers, regardless of their background or characteristics. Rasch analysis is a mathematical method to calibrate linear logit measures of item difficulty and person ability from ordinal data. The Rasch model posits that an individual's response probability is a composite of both their "person ability" and the "item difficulty"^12^. In this context, "person ability" refers to the extent to which participants express a positive attitude, while "item difficulty" pertains to the level of attitude required for each item. The Rasch model further proposes that the response probabilities of each individual to each item can be modeled as a logistic function of their latent attitude trait. This model provides a scientific framework for analyzing the relationship between individual attitudes and item characteristics and offers valuable insights into the underlying mechanisms governing response behavior in attitudinal research.

To test whether the data could fit the Rasch model, fit statistics, e.g., information-weighted fit statistics (infit) mean square (MNSQ) and outlier-sensitive fit statistics (outfit) MNSQ were used. An item with infit or outfit MNSQ out of the 0.7 to 1.5 range was considered a misfit^13^. We used a stricter MNSQ range of 0.65 to 1.3 to produce higher reliability and separation coefficient.

A Wright map is used to observe to what extent the item positions matched the person positions. The plot item difficulty and the individual’s abilities along its continuum on the same axis of the logits allow the evaluation of the fit of the item difficulties matched to the abilities of the individuals.

To test which item, differs between two different times (2019 data vs.1996 data), the differential item functioning (DIF) across two times was employed. DIF indicates that one group of respondents scores better than another group of respondents on an item (after adjusting for the overall scores of the respondents). This could mean the item has its usual difficulty for one group but is more difficult (or easier) than usual for the other. A DIF contrast ≥ 0.64 indicated a substantial DIF contrast^14^.

Finally, reliability was evaluated using the person separation index (comparable to Cronbach’s alpha). Person separation index denotes how well the test is able to differentiate among groups of respondents with different levels of positive attitude. An acceptable value for separation is at least 2. Item reliability was assessed using the item separation index. The separation value was less than 3 and the item reliability was less than 0.9, implying that the sample was insufficient to endorse construct validity or a difficulty existed with the item hierarchy of the instrument^14^.

All analyses were conducted using IBM SPSS for Windows, Version 22 (Chicago, IL, USA), and the Polytomous Rasch rating scale model using Winsteps Measurement Software, Version 5.3.4.0 (Winsteps® Rasch Measurement, 2022).

### Ethics approval and consent to participate

The study was conducted according to the guidelines of the Declaration of Helsinki and approved by the Institutional Review Board (or Ethics Committee) of the Faculty of Medicine, Chiang Mai University (study code: PSY-2566-09446 and date of approval, 14th March 2023).

### Informed consent

Informed consent was obtained from all individual participants included in the study.

## Results

### Demographic data and ATP-30 score

Of 360 participants, the mean age of participants in 1996 and 2019 was 22.67 years old (SD = 1.30), and 22.72 years old (SD = 1.3), respectively. The ratio of male/female was 285/337 in 1996, whereas 62/54 in 2019. The mean total score of ATP-30 in 1996 and 2019 was 100.60 (SD = 9.0) and 119.86(SD = 13.0), respectively. The mean difference was 19.26 (SD = 1.2) (t (*df* 316.79) = 16.29, p-value < 0.001).

Comparing the two groups, twenty-four items were scored higher in 2019, of these, nineteen items were statistical significantly (p < 0.05), whereas 6 items were scored lower in 2019 but non-significantly (Table 1).

**Table 1:** Mean and Standard deviation (SD) of the total score and individual item score of the ATP-30 in 1996 and 2019.

ATP-30	Year 1996		Year 2019		t-test	p-value
Item description	Mean	SD	Mean	SD		
1 (NO) little use of medial training	2.1	0.928	4.85	0.357	40.417	< 0.001
2 (NO) talk a lot but do very little	2.74	1.097	4.37	0.801	16.027	< 0.001
3 (NO) little more than prisons	3.01	1.202	4.45	0.819	13.322	< 0.001
4 like to be a psychiatrist	2.9	0.999	2.85	1.087	− 0.396	0.692
5 Accept the efficacy of psychotherapy	3.3	0.927	3.61	0.963	2.908	0.004
6 (NOT) running away from real medicine	3.31	1.13	4.61	0.603	14.160	< 0.001
7 (NO) talk about nothing but sex^†^	3.97	0.947	4.83	0.408	11.980	< 0.001
8 (NO) no strong evidence that it is effective	3.36	1.091	4.6	0.569	14.164	< 0.001
9 Increases our understanding patients	3.92	0.873	3.88	0.908	− 0.307	0.759
10 Psychiatric training has been valuable	3.33	0.858	3.4	0.893	0.752	0.453
11 is a respected branch of medicine	4.05	0.783	3.96	0.887	− 0.961	0.337
12 Psychiatric illness deserves attention	3.42	1.172	3.4	1.063	− 0.152	0.879
13 (NOT) has very little scientific information	3.36	1.051	3.92	1.007	4.820	< 0.001
14 Most psychiatric patients improve	3.5	0.814	4.03	0.832	5.703	< 0.001
15 At least as stable as the average doctor^†^	3.06	0.954	4.18	0.947	10.532	< 0.001
16 (NO) treatment causes patients to worry	3.52	1.084	3.74	1.09	1.857	0.064
17 (NOT) experience less satisfaction than other specialists	3.39	1.021	4.07	1.068	5.720	< 0.001
18 Interested to unravel cause	3.62	0.978	4.12	0.912	4.820	0.020
19 (NO) very little that psychiatrists can do^†^	3.53	1.044	4.55	0.825	10.069	< 0.001
20 Psychiatric hospitals have a specific role	3.81	0.889	3.8	1.045	− 0.068	0.946
21 Among specialties, psychiatry is (NOT) to be excluded	3.08	1.001	3.26	1.312	1.283	0.200
22 (NOT) hard to think of psychiatrists as equal	3.20	1.098	4.21	1.01	8.596	< 0.001
23 The most important part of the curriculum^†^	3.49	0.967	4.17	0.879	6.645	< 0.001
24 Psychiatry is so (NOT) unscientific	3.36	1.009	4.47	0.831	11.125	< 0.001
25 Treatment has become quite effective	3.55	0.843	3.9	0.839	3.709	0.041
26 (NOT) really just vague speculations	2.97	0.852	3.8	0.954	7.978	< 0.001
27 Patients are just as human as other people	3.65	1.201	3.57	1.196	− 0.625	0.532
28 Practice of psychiatry allows real relationships	4.10	0.672	4.24	0.881	1.477	0.141
29 Patients are often more interesting to work with	3.08	0.863	3.17	0.98	0.844	0.399
30 Psychiatry is (NOT) amorphous	2.91	1.117	3.87	1.064	7.902	< 0.001
Total score	100.60	9.02	119.86	13.02	16.29	< 0.001

*ATP* attitude to psychiatry.

^†^Females scored significantly higher than males (p < 0.05).

No differences were noted in each item score between males and females in 2019, except for items 7 and 12. The same was true in 1996, except for item 20.

#### Rasch analysis results

The logit, Item fit statistics, DIF, and reliability are presented as described below.

The item statistics including mean logit, standard error, infit MNSQ, outfit MNSQ, DIF measure, and standard error between 1996 and 2019, and the DIF contrast are shown in Table 2. The mean logit of the ATP-30 for the whole sample was 0.37(SE = 0.04) for students of 1996, and 1.26(SE = 0.04) for students of 2019, t (319) = 17.6, p < 0.001. The MNSQ for infit ranged for the overall sample from 0.67 to 1.44. The MNSQ for outfit ranged for the overall sample from 0.62 to 1.47. The person/item reliability was 0.86/0.96.

**Table 2:** Overall item difficulty (measure) and DIF contrast.

ATP-30 item	Measure	SE	Infit MNSQ	Outfit MNSQ	Year 1996		Year 2019		DIF contrast
					DIF Measure	DIF S.E	DIF Measure	DIF S.E	
1 (NOT)so little use of medical training	0.09	0.05	0.88	0.67	0.70	0.09	− 0.78	0.13	**− 1.48**
2 (NOT) talk a lot but do very little	− 0.03	0.06	0.94	1.01	0.42	0.09	− 0.24	0.09	**− 0.66**
3 (NOT) little more than prisons	− 0.09	0.06	0.72	0.69	0.27	0.09	− 0.28	0.09	− 0.55
4 likes to be a psychiatrist	1.18	0.06	1.2	1.19	0.64	0.12	1.71	0.08	**1.07**
5 accept the efficacy of psychotherapy	0.48	0.07	1.14	1.24	0.30	0.12	0.79	0.09	0.49
6 (NOT) running away from real medicine	− 0.42	0.06	0.84	0.83	− 0.03	0.09	− 0.73	0.11	**− 0.70**
7 (NOT) talk about nothing but sex	− 0.52	0.08	0.83	0.77	− 0.13	0.11	− 0.99	0.16	**− 0.87**
8 (NOT) no strong evidence that it is effective	− 0.51	0.07	0.73	0.67	− 0.07	0.1	− 0.86	0.11	− **0.79**
9 Increases our understanding patients	− 0.03	0.07	1.2	1.21	− 0.58	0.14	0.4	0.09	**0.98**
10 psychiatric training has been valuable	0.49	0.07	1.18	1.21	0.06	0.13	0.91	0.09	**0.85**
11 is respected branch of medicine	− 0.47	0.08	1.08	1.11	− 1.13	0.15	− 0.01	0.1	**1.12**
12 Psychiatric illness deserves attention	0.37	0.06	1.44	1.47	− 0.2	0.11	0.67	0.07	**0.86**
13 (NOT) has very little scientific information	0.07	0.06	0.99	1.06	0.04	0.11	0.25	0.08	0.20
14 Most psychiatric patients improve	− 0.17	0.08	0.87	0.86	− 0.02	0.13	− 0.14	0.11	− 0.11
15 At least as stable as the average doctor	0.02	0.06	0.88	0.90	0.41	0.10	− 0.13	0.09	− 0.54
16 treatment (NOT) causes patients to worry	0.23	0.06	1.17	1.28	− 0.08	0.11	0.59	0.08	**0.67**
17 (NOT) get less satisfaction than other specialists	− 0.06	0.06	0.94	1.04	− 0.04	0.1	0.07	0.08	0.10
18 Interesting to unravel cause	− 0.24	0.07	0.95	0.93	− 0.23	0.12	− 0.12	0.09	0.12
19 (NOT) very little that psychiatrists can do	− 0.38	0.07	0.74	0.68	− 0.10	0.09	− 0.6	0.11	− 0.49
20 Psychiatric hospitals have a specific	0.08	0.07	1.27	1.39	− 0.4	0.12	0.51	0.09	**0.91**
21 Among specialties, psychiatry is (NOT) to be excluded	0.73	0.06	1.28	1.26	0.27	0.10	0.97	0.07	**0.70**
22 (NOT) hard to think of psychiatrists as equal	− 0.38	0.06	0.84	0.91	− 0.13	0.1	− 0.41	0.09	− 0.28
23 The most important part of the curriculum	− 0.49	0.07	0.9	0.91	− 0.28	0.11	− 0.5	0.1	− 0.22
24 Psychiatry is (NOT) so unscientific	− 0.39	0.07	0.67	0.62	0.00	0.10	− 0.62	0.1	− 0.62
25 Treatment has become quite effective	− 0.29	0.08	0.9	0.9	− 0.38	0.14	− 0.1	0.1	0.28
26 (NOT) really just vague speculations	− 0.09	0.07	0.84	0.83	0.19	0.12	− 0.06	0.09	− 0.25
27 Patients are just as human as other people	0.3	0.06	1.37	1.45	− 0.25	0.10	0.61	0.07	**0.86**
28 Practice of psychiatry allows real relationships	− 0.37	0.09	0.97	0.96	− 0.61	0.15	− 0.09	0.11	0.52
29 Patients are often more interesting to work with	0.73	0.07	1.23	1.23	0.14	0.13	1.00	0.09	**0.86**
30 Psychiatry is (NOT) so amorphous	0.17	0.06	0.85	0.92	0.31	0.1	0.25	0.08	− 0.06

Significant values are in bold.

*ATP* attitude toward psychiatry, *DIF* differential item functioning, *MNSQ* mean square.

DIF items were answered differently by the two groups even after controlling the total score. Fifteen items with |DIF contrast|≥ 0.64 were detected. DIF contrasts between the two groups are displayed in Table 2.

All these DIF items can be grouped into five components using principal component analysis. First, poor image (Q1, 8, 6,7, and 2), second, preference (Q4, 21, and 29), third, hospital and training (Q20 and 10), fourth, patients and illness (Q27, 11, and 12), and the fifth, causes to worry (Q16).

The Wright map shows that the mean person ability in 2019 was higher than those in 1996, suggesting that the attitude towards psychiatry of the students in 2019 was higher than that of students in 1996. Many different hierarchical items were noted between 1996 and 2019.

**Figure 1:**
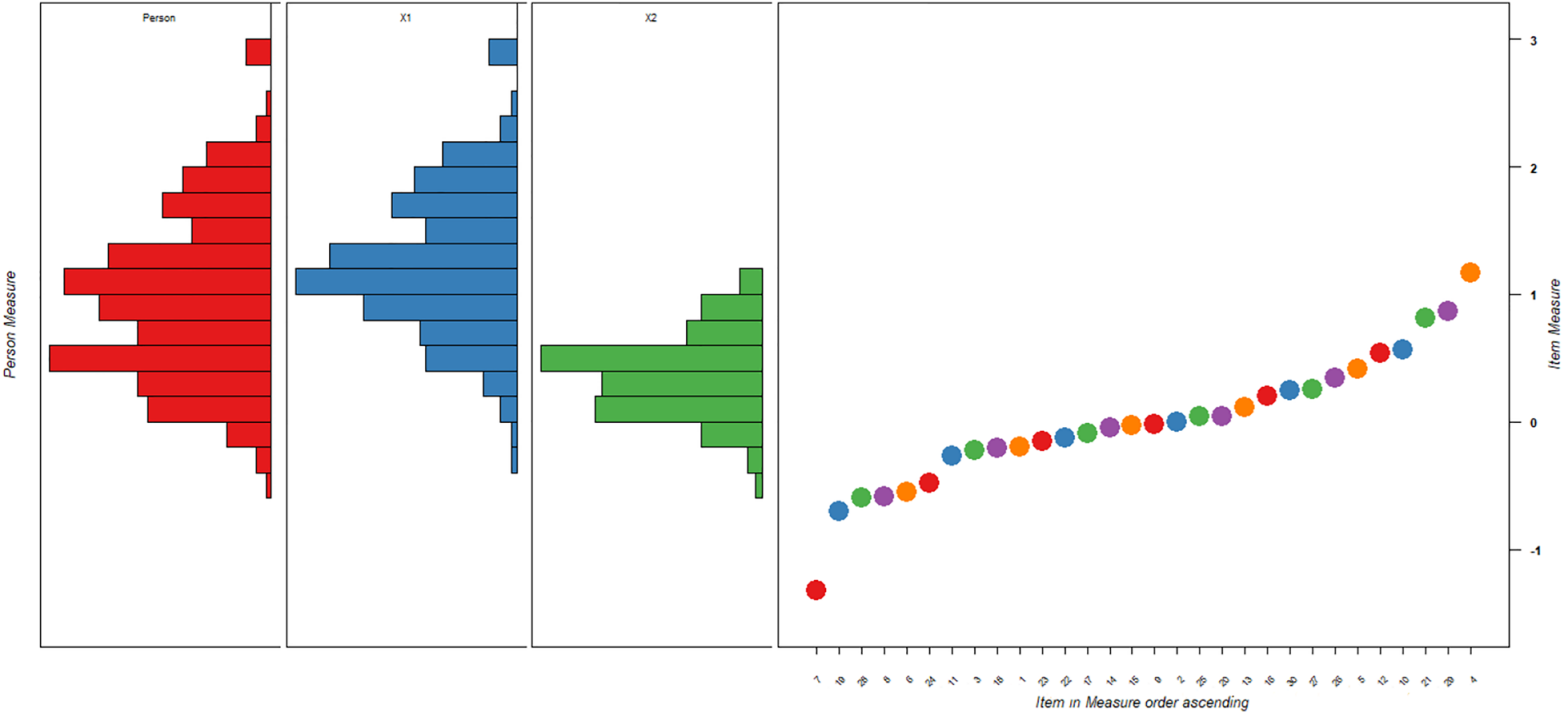
Wright Map depicting the distribution of person and item between 1996 and 2019. X1 = 2019 group, X2 = 1996 group, the item (on the right), item 7 is the easiest; item 4 is the hardest. The mean ability of the 2019 group is higher than the 1996 group. Colored rounds represent each item located ascendingly in measure (logit).

The visualizing Wright map (Fig. 1) shows that for the whole group (combining data in 1996 and 2019), the mean person ability of 2019(X1) was higher than that in 1996(X2), suggesting that the attitude towards psychiatry of the students of 2019 was higher than that of students in 1996.

In terms of the item difficulty, Q7: “Psychiatrists seem to talk about nothing but sex” indicated that this was the easiest item (logit = − 0.52(0.08)) (the most positive attitude, and everybody did not endorse this item), whereas Q4: “I would like to be a psychiatrist” was the most difficult item (logit = 1.18 (0.06)) (the least positive attitude, less than one-half of all respondents endorsed it). For the remaining items, see Table 2.

**Figure 2:**
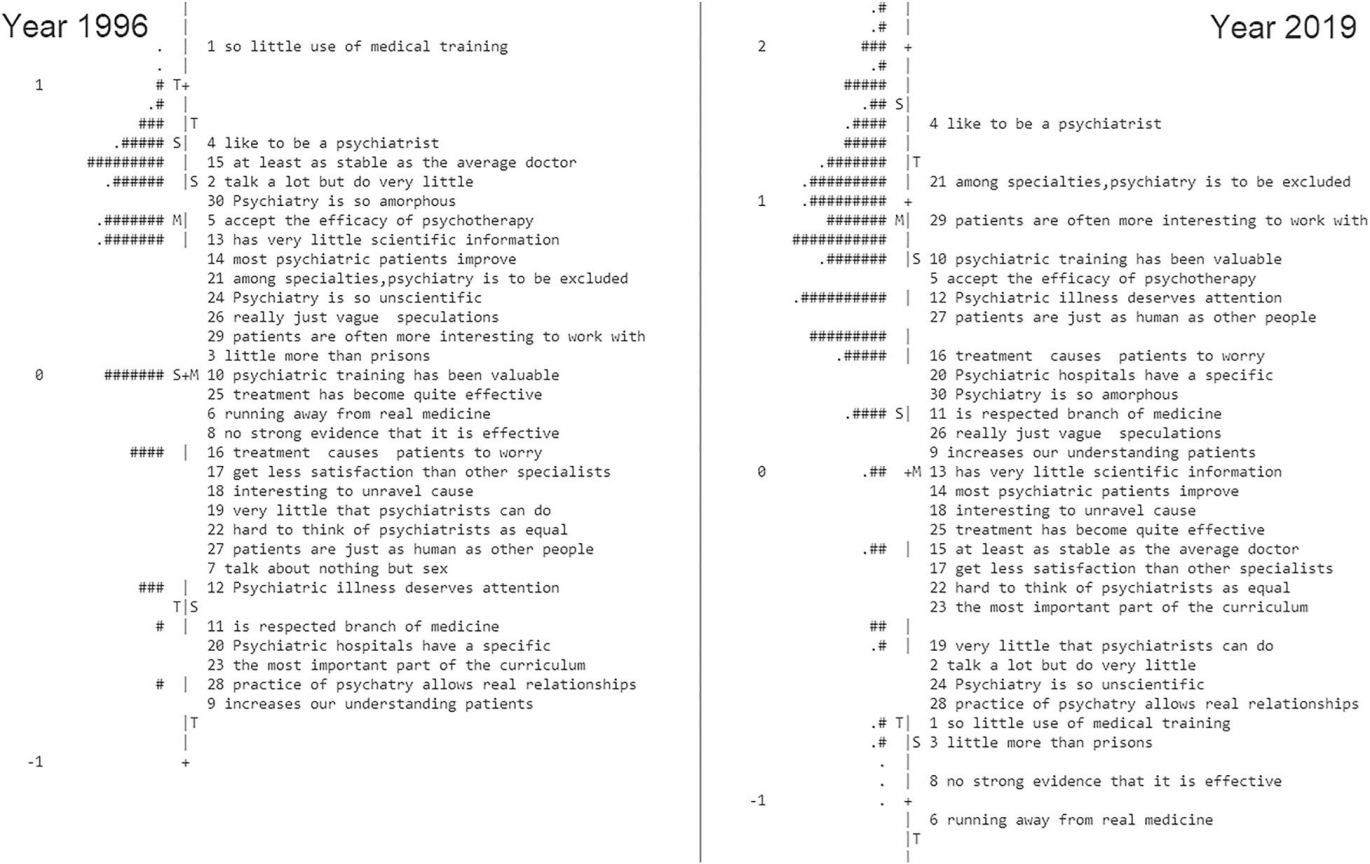
Wright Map depicting the item hierarchy between 1996 and 2019. For 1996, each "#" represents 5 persons, each "." represents 1 to 4 persons. For 2019, each "#" represents 2 persons, each "." represents 1 person.

In a separate analysis between 1996 and 2019 (Fig. 2), item fit statistics, the MNSQ for infit/outfit, ranged from 0.78 to 1.59 among the participants of 1996, and from 0.65 to 1.43 for infit/outfit among the participants of 2019, indicating that all items sufficiently fit the model (MNSQ < 2). However, the reliability of the data from 1996 was lower than that in 2019 (the person/item reliability was 0.67/0.93 and 0.89/0.99 for the sample of 1996 and 2019, respectively.) The individual Wright map shows the much different positions of some items, e.g., Q1, Q15, Q2, and so on, suggesting DIF between the groups.

**Figure 3:**
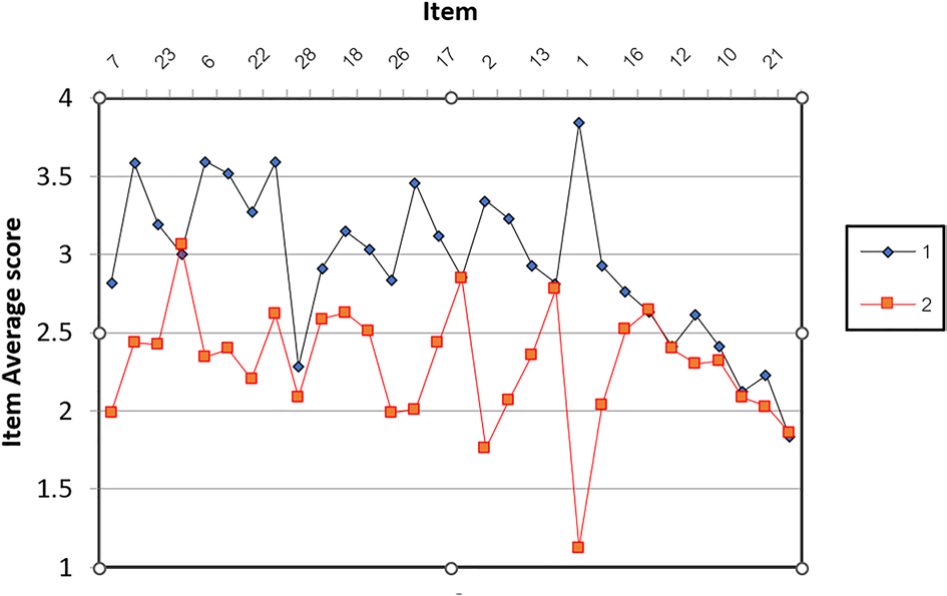
The plot illustrating each ATP item’s average score comparing 1996 and 2019. 1 = 2019, 2 = 1996, * = average.

The plot of item average scores between 1996 and 2019 is shown in Fig. 3. Almost all items scored higher in 2019 than in 1996.

In examining the misfitted items and persons, 34 misfitting persons having MNSQ > 2 were identified and removed. The final data included 104 persons for the 1996 group and 222 persons for the 2019 group. Fifteen items (Q1, Q4, Q5, Q9, Q10, Q11, Q12, Q13, Q16, Q18, Q20, Q21, Q27, Q28, and Q29) were the misfitting items if a stricter MNSQ cut-off of 1.3 was applied. The remaining items were considered valid and useful to be compared between two groups, which included Q23, Q2, Q17, Q25, Q22, Q30, Q26, Q14, Q3, Q7, Q6, Q8, Q19, and Q24 (Table S1). Item fit statistics, the MNSQ for infit/outfit, ranged from 0.65 to 1.30. The person/item reliability was 0.85/0.92. Notably, 12/15 (80%) of the misfitting items were DIF items.

Table 3 shows a 15-item ATP after removing the DIF items (a comparison without item biases).

**Table 3:** Mean and Standard deviation (SD) of the total score and individual item score of the ATP in 1996 and 2019 after removing DIF items (ATP-15).

ATP-15	Year 1996		Year 2019		t-test	p-value
Item description	Mean	SD	Mean	SD		
3 (NO)little more than prisons	3.01	1.202	4.45	0.819	− 13.32	< 0.001
5 accept the efficacy of psychotherapy	3.30	0.927	3.61	0.963	− 2.91	0.004
13 (NOT) has very little scientific information	3.36	1.051	3.92	1.007	− 4.82	< 0.001
14 Most psychiatric patients improve	3.50	0.814	4.03	0.832	− 5.70	< 0.001
15 At least as stable as the average doctor	3.06	0.954	4.18	0.947	− 10.53	< 0.001
17 (NOT) get less satisfaction than other specialists	3.39	1.021	4.07	1.068	− 5.72	< 0.001
18 interesting to unravel cause	3.62	0.978	4.12	0.912	− 4.82	0.020
19 (NO) very little that psychiatrists can do	3.53	1.044	4.55	0.825	− 10.07	< 0.001
22 (NOT) hard to think of psychiatrists as equal	3.20	1.098	4.21	1.01	− 8.60	< 0.001
23 The most important part of the curriculum	3.49	0.967	4.17	0.879	− 6.65	< 0.001
24 Psychiatry is so (NOT) unscientific	3.36	1.009	4.47	0.831	− 11.13	< 0.001
25 Treatment has become quite effective	3.55	0.843	3.90	0.839	− 3.71	0.041
26 (NOT) really just vague speculations	2.97	0.852	3.80	0.954	− 7.98	< 0.001
28 Practice of psychiatry allows real relationships	4.10	0.672	4.24	0.881	− 1.48	0.141
30 Psychiatry is (NOT) amorphous	2.91	1.117	3.87	1.064	− 7.90	< 0.001
Total score	50.35	5.91	61.55	8.52	− 14.47	< 0.001

*ATP* attitude toward psychiatry, *DIF* differential item functioning.

Principal component analysis was conducted on the 15 unbiased items, resulting in the identification of three components. The first component represented an unscientific attitude toward psychiatry (Q30, Q26, Q17, Q22, Q24, and Q19), while the second component indicated a negative attitude toward psychiatric methods (Q3, Q7, Q8, Q6, and Q2). The third component reflected the efficacy of psychiatric treatment (Q25, Q23, Q14, and Q15) (Supplement File S2).

The mean total score and individual scores of each item were higher in 2019 than that in 1996. Eleven items were found highly significantly different (p < 0.01). With stricter criteria (the MNSQ infit/outfit ranged from 0.65 to 1.30).

## Discussion

The study aimed to examine the attitude of medical students toward psychiatry over two extended periods resulting in two main findings. First, the overall attitude scores were higher among the medical students in the recent year than those in the past two decades; without the items with DIF, the scores of all items were higher in the recent group. Second, regarding the manner of the items, one-half of the scale functioned differently for groups.

The outcome is in line with other related studies if we compare the level of attitude using the same ATP-30. A study in Lebanon discovered that 95.1% had a positive attitude toward psychiatry with an average ATP-30 score of 111.95(SD = 12.55), which is comparable with the present study (mean 119.86(SD = 13.0)^15^. The same is true for higher socio-economic countries such as Germany and Switzerland^16^. These findings supported our hypothesis that the overall score of attitudes of the students at the recent time should be higher than that in the past. This could be due to many positive issues during the past 20 years, as mentioned in the introduction.

In terms of item difficulty, Q4: I would like to be a psychiatrist is still the hardest item, whereas “Q7: Psychiatrists seem to talk about nothing but sex” is the easiest. This proved our hypothesis wrong regarding the intention to become a psychiatrist during the recent period. These results are inconsistent compared with other related studies^17,18^. Although the fact that Q4 is the most difficult should make more sense, this was not always the case because a study revealed that the choice of a psychiatrist career is influenced by many factors, such as personality traits and characteristics, career aspects, stigma towards mental illness, student values and personal needs^2,8,19^.

Regarding the DIF items, we cannot be certain that these items are biased, but we can be assured that such items functioned differently for groups. In principle, we should be able to offer hypotheses to explain why some items exhibit DIF. However, offering clear explanations is often difficult. We can say that two groups of people endorsed differently on the 15 items. Recall that 80% of the DIF comprised misfitting items, which are usually individual items with aberrant, unexpected, unpredictable response patterns^20^. When delving deeper into their contents, these misfitting and DIF items seem to be “too” pessimistic, for example, Q1. Psychiatry is unappealing because it makes so little use of medical training and Q8: The practice of psychotherapy is fraudulent. On the other hand, some items seem to be “too” optimistic,” for example, Q11. Psychiatry is a respected branch of medicine; Q29 Psychiatric patients are often more interesting to work with than other patients and Q4. I would like to be a psychiatrist. The extreme stimuli of these items may be prone to erratic responses from the respondents.

If we look at the content of the fitting and nonDIF items, they seem to be more neutral, which might help draw better attention from the respondents and allow a good judgment before endorsing it. For example, Q5: It is quite easy for me to accept the efficacy of psychotherapy and Q17. Psychiatrists get less satisfaction from their work than other specialists. All these items are related to the esteem and value of psychiatry and treatment efficacy.

When comparing only valid items, fitting and nonDIF, scores are significantly higher in the recent group than in the past group. However, it would be difficult to pinpoint what factors are specific to the increased attitude. The authors speculate that these changes should be related to the students’ better understanding of psychiatry, especially the burgeoning of scientific evidence, treatment efficacy, and successful teaching methods. However, it should be noted that the impact of successful teaching methods cannot be conclusively determined, as no assessments were conducted at the start of the clerkship for comparison.

### Implications of the study

Increasing students’ attitudes can be promoted in several ways depending on the various contexts, e.g., medical curriculum, teaching methods, and opportunity for clinical exposure and experience in psychiatry. Attention can be paid to the low score item after misfitting items and persons have been removed. Otherwise, the true answers may be interfered with by the aberrant items and responses. Some ATP items should be revised or removed. In line with the related study, we found some items that are not suitable for the scale, e.g., items 12 and 29^21^. Some details liable to misfitting and DIF items should be revised. For example, Q4: I would like to be a psychiatrist, which can be toned down to “there is a possibility that I would choose to be a psychiatrist” which might be more neutral and pull a better positive response.

Demonstrating a sufficient unidimensional construct of the attitude would be more significant than a multidimensional construct, as it would provide assurance that the total score of the scale can be reliably used and compared across studies that use the same scale.

### Strength and limitations

This research is probably the first to compare medical students’ attitudes over two long periods. At least it helps us to realize what has changed in different locations and times. Some limitations to be addressed included the samples of both times being recruited in a convenient method. Thus, it may not represent all Chiang Mai University and Thai medical students. The lack of comparison between the beginning and the end of the clerkship, we cannot establish how the clerkship influence the differences of the students’ attitudes. Response bias due to social desirability cannot be excluded using self-report measurement.

In conclusion, the attitudes towards psychiatry of the recent fifth-year medical students were significantly higher than those in the past. Even though the item scores of all the ATP escalated, the intention to be a psychiatrist did not increase as expected. Item and person misfits and DIF items should be identified and removed to compare more fairly between groups. Implications of the study are discussed, and further study regarding improving the items measuring attitude should be encouraged.

## Supplementary Information

Supplementary Information.

## Data Availability

The datasets used and/or analyzed during the current study are available from the corresponding author on reasonable request.
