# Author Correction: Medical students’ attitude towards psychiatry: a comparison of past and present

**DOI:** 10.1038/s41598-023-37291-x

**Published:** 2023-06-20

**Authors:** Punjaree Wiriyacosol, Awirut Oon‑arom, Chawisa Suradom, Nahathai Wongpakaran, Tinakon Wongpakaran

**Affiliations:** 1 Department of Psychiatry, Faculty of Medicine, Chiang Mai University, Chiang Mai, 50200 Thailand; 2 Psychotherapy Unit and Geriatric Psychiatry Unit, Department of Psychiatry, Faculty of Medicine, Chiang Mai University, 110 Intawaroros Rd., T. Sriphum, A. Muang, Chiang Mai, 50200 Thailand

Correction to: *Scientific Reports* 10.1038/s41598-023-35797-y, published online 29 May 2023

The original version of this Article contained an error in the Abstract.

“Negatively worded items, e.g., no strong evidence indicating effectiveness, became easier to score items (increased positive attitude) whereas some positively worded items, e.g., I would like to be a psychiatrist. Nine items, became more difficult (less positive attitude) comparing between 1996 and 2019.”

now reads:

“Negatively worded items, e.g., no strong evidence indicating effectiveness, became easier to score items (increased positive attitude) whereas some positively worded items, e.g., I would like to be a psychiatrist, became more difficult (less positive attitude) comparing between 1996 and 2019.”

The original Article has been corrected.

