# Supplementary material for: Medical students’ attitude towards psychiatry: a comparison of past and present
Source: Sci Rep. 2023 May 29;13:8714. doi: 10.1038/s41598-023-35797-y (PMC10227019; doi:10.1038/s41598-023-35797-y)
Supplement: Supplementary file 1 — Supplementary Information. [file 41598_2023_35797_MOESM1_ESM.zip › Supplement file S2.docx]

Supplement file S2

| **KMO and Bartlett's Test** | | |
| --- | --- | --- |
| Kaiser-Meyer-Olkin Measure of Sampling Adequacy. | | .919 |
| Bartlett's Test of Sphericity | Approx. Chi-Square | 2172.969 |
|  | df | 105 |
|  | Sig. | .000 |

| **Communalities** | | |
| --- | --- | --- |
|  | Initial | Extraction |
| 2 talk a lot but do very little | 1.000 | .435 |
| 3 little more than prisons | 1.000 | .654 |
| 6 running away from real medicine | 1.000 | .592 |
| 7 talk about nothing but sex | 1.000 | .568 |
| 8 no strong evidence that it is effective | 1.000 | .632 |
| 14 most psychiatric patients improve | 1.000 | .604 |
| 15 at least as stable as the average doctor | 1.000 | .455 |
| 17 get less satisfaction than other specialists | 1.000 | .545 |
| 19 very little that psychiatrists can do | 1.000 | .634 |
| 22 hard to think of psychiatrists as equal | 1.000 | .574 |
| 23 the most important part of the curriculum | 1.000 | .557 |
| 24 Psychiatry is so unscientific | 1.000 | .662 |
| 25 treatment has become quite effective | 1.000 | .683 |
| 26 really just vague speculations | 1.000 | .577 |
| 30 Psychiatry is so amorphous | 1.000 | .643 |
| Extraction Method: Principal Component Analysis. | | |

| **Total Variance Explained** | | | | | | | | | |
| --- | --- | --- | --- | --- | --- | --- | --- | --- | --- |
| Component | Initial Eigenvalues | | | Extraction Sums of Squared Loadings | | | Rotation Sums of Squared Loadings | | |
|  | Total | % of Variance | Cumulative % | Total | % of Variance | Cumulative % | Total | % of Variance | Cumulative % |
| 1 | 6.202 | 41.349 | 41.349 | 6.202 | 41.349 | 41.349 | 3.147 | 20.983 | 20.983 |
| 2 | 1.473 | 9.820 | 51.169 | 1.473 | 9.820 | 51.169 | 3.101 | 20.671 | 41.654 |
| 3 | 1.140 | 7.599 | 58.768 | 1.140 | 7.599 | 58.768 | 2.567 | 17.114 | 58.768 |
| 4 | .899 | 5.995 | 64.763 |  |  |  |  |  |  |
| 5 | .702 | 4.679 | 69.442 |  |  |  |  |  |  |
| 6 | .625 | 4.164 | 73.606 |  |  |  |  |  |  |
| 7 | .584 | 3.893 | 77.500 |  |  |  |  |  |  |
| 8 | .569 | 3.796 | 81.295 |  |  |  |  |  |  |
| 9 | .475 | 3.165 | 84.461 |  |  |  |  |  |  |
| 10 | .469 | 3.126 | 87.587 |  |  |  |  |  |  |
| 11 | .450 | 3.001 | 90.587 |  |  |  |  |  |  |
| 12 | .405 | 2.702 | 93.289 |  |  |  |  |  |  |
| 13 | .349 | 2.328 | 95.617 |  |  |  |  |  |  |
| 14 | .336 | 2.243 | 97.860 |  |  |  |  |  |  |
| 15 | .321 | 2.140 | 100.000 |  |  |  |  |  |  |
| Extraction Method: Principal Component Analysis. | | | | | | | | | |

| **Component Matrix^a^** | | | |
| --- | --- | --- | --- |
|  | Component | | |
|  | 1 | 2 | 3 |
| 24 Psychiatry is so unscientific | .803 |  |  |
| 19 very little that psychiatrists can do | .783 | .143 |  |
| 30 Psychiatry is so amorphous | .693 |  | -.394 |
| 8 no strong evidence that it is effective | .693 | -.389 |  |
| 22 hard to think of psychiatrists as equal | .683 | .114 | -.307 |
| 3 little more than prisons | .660 | -.415 | .217 |
| 6 running away from real medicine | .659 | -.395 |  |
| 26 really just vague speculations | .626 |  | -.428 |
| 14 most psychiatric patients improve | .604 | .471 | .137 |
| 17 get less satisfaction than other specialists | .600 | .145 | -.405 |
| 7 talk about nothing but sex | .599 | -.436 | .140 |
| 15 at least as stable as the average doctor | .593 |  | .306 |
| 23 the most important part of the curriculum | .552 | .392 | .314 |
| 25 treatment has become quite effective | .526 | .471 | .429 |
| 2 talk a lot but do very little | .488 | -.356 | .264 |
| Extraction Method: Principal Component Analysis. | | | |
| a. 3 components extracted. | | | |

| **Rotated Component Matrix^a^** | | | |
| --- | --- | --- | --- |
|  | Component | | |
|  | 1 | 2 | 3 |
| 30 Psychiatry is so amorphous | .749 | .223 | .180 |
| 26 really just vague speculations | .725 | .205 |  |
| 17 get less satisfaction than other specialists | .709 | .119 | .167 |
| 22 hard to think of psychiatrists as equal | .682 | .222 | .244 |
| 24 Psychiatry is so unscientific | .593 | .377 | .410 |
| 19 very little that psychiatrists can do | .506 | .364 | .496 |
| 3 little more than prisons | .177 | .767 | .186 |
| 7 talk about nothing but sex | .194 | .722 |  |
| 8 no strong evidence that it is effective | .356 | .703 | .105 |
| 6 running away from real medicine | .311 | .697 | .101 |
| 2 talk a lot but do very little |  | .637 | .164 |
| 25 treatment has become quite effective |  | .102 | .815 |
| 23 the most important part of the curriculum | .177 | .139 | .712 |
| 14 most psychiatric patients improve | .357 |  | .688 |
| 15 at least as stable as the average doctor | .158 | .379 | .535 |
| Extraction Method: Principal Component Analysis.  Rotation Method: Varimax with Kaiser Normalization. | | | |
| a. Rotation converged in 5 iterations. | | | |

| **Component Transformation Matrix** | | | |
| --- | --- | --- | --- |
| Component | 1 | 2 | 3 |
| 1 | .628 | .593 | .504 |
| 2 | .175 | -.739 | .651 |
| 3 | -.758 | .321 | .567 |
| Extraction Method: Principal Component Analysis.  Rotation Method: Varimax with Kaiser Normalization. | | | |
