# Supplementary material for: Medical students’ attitude towards psychiatry: a comparison of past and present
Source: Sci Rep. 2023 May 29;13:8714. doi: 10.1038/s41598-023-35797-y (PMC10227019; doi:10.1038/s41598-023-35797-y)
Supplement: Supplementary file 1 — Supplementary Information. [file 41598_2023_35797_MOESM1_ESM.zip › Supplement file Table S1.docx]

Supplement file

| **Table S1.** The INFIT and OUTFIT mean square of the ATP-15 items. | | | | |
| --- | --- | --- | --- | --- |
| item  NUMBER | MEASURE | MODEL  S.E. | INFIT  MNSQ | OUTFIT  MNSQ |
| 23 | -0.24 | 0.08 | 1.2 | 1.3 |
| 2 | 0.25 | 0.07 | 1.2 | 1.25 |
| 17 | 0.21 | 0.07 | 1.18 | 1.23 |
| 25 | 0.02 | 0.09 | 1.19 | 1.2 |
| 15 | 0.3 | 0.07 | 1.09 | 1.14 |
| 22 | -0.12 | 0.07 | 1.02 | 1.12 |
| 30 | 0.54 | 0.07 | 1.06 | 1.1 |
| 26 | 0.32 | 0.08 | 1.05 | 1.04 |
| 14 | 0.11 | 0.09 | 1.04 | 1.04 |
| 3 | 0.14 | 0.07 | 0.84 | 0.95 |
| 7 | -0.41 | 0.09 | 0.86 | 0.92 |
| 6 | -0.26 | 0.07 | 0.86 | 0.88 |
| 8 | -0.37 | 0.08 | 0.79 | 0.77 |
| 19 | -0.25 | 0.08 | 0.77 | 0.72 |
| 24 | -0.22 | 0.08 | 0.72 | 0.65 |
